# Predicting mortality for Covid-19 in the US using the delayed elasticity method

**DOI:** 10.1038/s41598-020-76490-8

**Published:** 2020-11-30

**Authors:** Luis Ángel Hierro, Antonio J. Garzón, Pedro Atienza-Montero, José Luis Márquez

**Affiliations:** 1 Department of Economics and Economic History, University of Seville, Avda. Ramón y Cajal, 1, 41018 Seville, Spain; 2 University Hospital Virgen del Rocio, Avda. Manuel Siurot s/n, 41013 Seville, Spain

**Keywords:** Infectious diseases, Public health

## Abstract

The evolution of the pandemic caused by COVID-19, its high reproductive number and the associated clinical needs, is overwhelming national health systems. We propose a method for predicting the number of deaths, and which will enable the health authorities of the countries involved to plan the resources needed to face the pandemic as many days in advance as possible. We employ OLS to perform the econometric estimation. Using RMSE, MSE, MAPE, and SMAPE forecast performance measures, we select the best lagged predictor of both dependent variables. Our objective is to estimate a leading indicator of clinical needs. Having a forecast model available several days in advance can enable governments to more effectively face the gap between needs and resources triggered by the outbreak and thus reduce the deaths caused by COVID-19.

## Introduction

Predictive models for the Covid-19 pandemic have been evolving as the available information has increased. For early predictions, purely predictive statistical models were applied^1–3^. However, as more information has become available, increasingly complex epidemiological models have been developed^4,5^. Both types of models are commonly used in epidemiology. The first type, predictive models, are built for the sole purpose of predicting the evolution of the variable under study (number of infections, deaths…) using past information from the same variable and employing probabilistic equations^6^, exponential smoothing methods^7^ or ARIMA techniques^7–9^. The latter, which are epidemiological models in the strict sense, are models which explain the spread of the disease, with most of them being of the “compartments” type, and which were developed following the works of Kermack and McKendrick^10–12^. These are intended to explain the spread of the infection in all its stages and to assess the effect of control measures such as social distancing or vaccination^13,14^.

In the early stages of the spread of a pandemic caused by a new virus, such as SARS-Cov-2, existing models evidence major problems in terms of prediction. Ioannidis, Cripps and Tanner^15^ have conducted a review of such problems to have arisen during the current pandemic. Causes of prediction failure include: lack of information on epidemiological parameters, ratios and constants, as well as the assumptions made when building epidemiological models, the use of exponential models, which have amplified errors, the need for a large number of observations in stochastic models… The Delayed Elasticity Method (DEM), which we applied to the initial stage of the Covid-19 pandemic in the US, is a new type of model in which we use the relationship between the death variable and the infected cases variable to forecast deaths from Covid-19. It is, in some respects, an intermediate model, which is predictive in the sense that its sole purpose is to predict, but which uses the relationship between deaths and infections for the estimation and, therefore, shares this feature with epidemiological models. Its advantages are that it needs relatively short time series, it defines a prediction window that other models do not define, added to which its predictive accuracy is very high.

## Methodology

We propose the following method (Delayed Elasticity Method-DEM). Using officially published data from the Johns Hopkins University CSSE^17^, we econometrically estimate the following equation:

1 $${\text{log}}\,(Deaths_{t} ) = \alpha + \beta\, {\text{log}}\,(Cases_{t - i} ) + \varepsilon_{t}$$

where i = 1, 2 …,10 are the number of delays of the explanatory variable, $Deaths_{t}$ is the total number of deaths up to day t, and $Cases_{t}$ is the number of cases detected up to date t.

The coefficient $\beta_{ - i}$ is what in economics is called elasticity and represents the relationship between the variation of the dependent and independent variables:

2 $$\beta_{ - i} = \frac{{\frac{{\Delta \widehat{{Deaths_{t} }}}}{{\widehat{{Deaths_{t} }}}}}}{{\frac{{\Delta Cases_{t - i} }}{{Cases_{t - i} }}}}$$

After estimating equations using different lags, we select the one with the best forecast performance (which minimizes forecasting errors). We calculate RMSE^16^ as an indicator of predictive accuracy. Other indicators, such as MAE, MAPE or SMAPE, can be used. RMSE is defined as follows:

3 $$RMSE = \sqrt {\frac{{\mathop \sum \nolimits_{t = T + 1}^{T + N} \left( {\hat{y} - y} \right)^{2} }}{N}}$$

where N is the number of out-of-sample observations, which we use to estimate the forecast performance of our estimate, $\hat{y}$ is the estimated value of the dependent variable, and $y$ is the actual value.

Finally, we select the estimate with the lagged explanatory variable that shows the lowest value in this indicator, which determines the prediction window, and we make the corresponding prediction in total values.

## Results

The estimation sample spans from 4/3/2020 to 29/3/2020 (26 observations). We left March 30, 31 as well as April 1 (3 observations) as out-of-sample observations in order to measure the forecast performance of the estimated model. Estimation is performed through the OLS estimator. We select the model including nine delays, since it shows the lowest RMSE value. The equation of the model that evidences the best forecast performance is the following:

4 $$\log \left( {\widehat{Deaths}_{t} } \right) = 0.3695 + 0.7839* {\text{log}}\,(Cases_{t - 9} )$$

The delayed elasticity, $\beta = 0.7839$, means that a 1% increase in the number of infected cases predicts a 0.78% increase in the number of deaths 9 days later. The estimate presents a high goodness of fit, with an R-square of 0.98.

**Figure 1:**
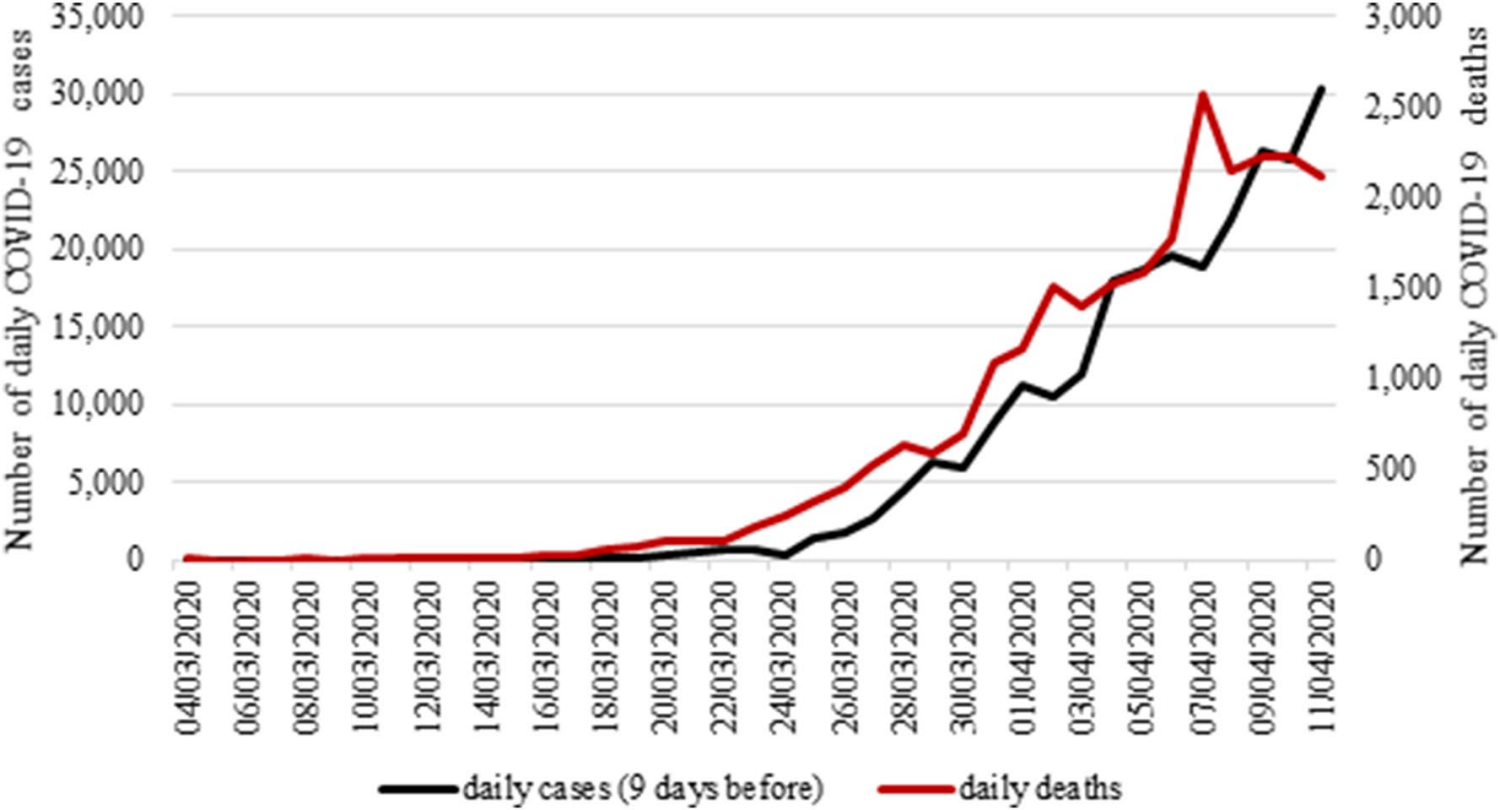
Evolution of daily confirmed COVID-19 deaths and 9-day delayed daily confirmed COVID-19 cases. *Source*: Authors’ own compilation and Johns Hopkins University CSSE (retrieved on 05/10/2020).

Figure 1 displays the evolution of daily new confirmed deaths and new confirmed cases nine days earlier.

Table 1 shows the number of actual and estimated deaths, as well as the errors for each time period. To obtain the total number of deaths, we carry out the following transformation:

5 $$\widehat{Deaths}_{t} = exp\,(\log \left( {\widehat{Deaths}_{t} } \right)$$

We also applied the DEM to other smaller areas, specifically to the State of California and the city of New York, using data for the same period, in order to test the robustness of the method. The equations which correspond to the best predictive accuracy for each case are the following (for the number of deaths in California and the number of deaths and infected cases in New York city, we add + 1 to the actual values in order to solve the missing value problem generated by observations that take the value 0 when we take logarithms):

**Table 1:** Actual versus estimated COVID-19 deaths and estimated error. *Source*: Authors’ own compilation and Johns Hopkins University CSSE (retrieved on 05/10/2020). Deaths(Est) values are rounded to integer values. Out-of-sample dates in bold.

Date	Cases	Deaths	Deaths (Est)	Error	Error rate
04/03/2020	107	11	13	2	15.62
05/03/2020	184	12	13	1	5.99
06/03/2020	237	14	13	− 1	9.15
07/03/2020	403	17	13	− 4	21.54
08/03/2020	519	21	13	− 8	36.49
09/03/2020	594	22	18	− 4	17.97
10/03/2020	782	28	22	− 6	21.79
11/03/2020	1147	33	33	0	1.46
12/03/2020	1586	43	42	− 1	1.74
13/03/2020	2219	51	56	5	10.62
14/03/2020	2978	58	86	28	48.77
15/03/2020	3212	70	105	35	50.33
16/03/2020	4679	97	160	63	64.48
17/03/2020	6511	132	195	63	47.38
18/03/2020	9165	191	216	25	13.22
19/03/2020	13,659	265	268	3	1.23
20/03/2020	20,026	364	362	− 2	0.49
21/03/2020	26,022	463	467	4	0.86
22/03/2020	34,824	573	608	35	6.05
23/03/2020	46,043	762	765	3	0.43
24/03/2020	56,620	1001	812	− 189	18.88
25/03/2020	68,654	1325	1091	− 234	17.69
26/03/2020	86,548	1733	1413	− 320	18.47
27/03/2020	105,179	2253	1847	− 406	18.01
28/03/2020	124,786	2886	2526	− 360	12.48
29/03/2020	143,715	3472	3409	− 63	1.81
30/03/2020	**165,728**	**4164**	**4186**	**22**	**0.53**
31/03/2020	**192,091**	**5249**	**5260**	**11**	**0.22**
01/04/2020	**217,910**	**6421**	**6548**	**127**	**1.98**
02/04/2020	**248,302**	**7924**	**7700**	− **224**	**2.82**
03/04/2020	**280,302**	**9316**	**8956**	− **360**	**3.86**
04/04/2020	**313,303**	**10,839**	**10,739**	− **100**	**0.92**
05/04/2020	**341,487**	**12,429**	**12,513**	**84**	**0.67**
06/04/2020	**371,672**	**14,199**	**14,307**	**108**	**0.76**
07/04/2020	**403,071**	**16,770**	**15,982**	− **788**	**4.70**
08/04/2020	**435,087**	**18,916**	**17,871**	− **1045**	**5.52**
09/04/2020	**469,735**	**21,144**	**20,064**	− **1080**	**5.11**
10/04/2020	**503,271**	**23,362**	**22,149**	− **1213**	**5.19**
11/04/2020	**532,628**	**25,481**	**24,536**	− **945**	**3.71**

- State of California:
  6 $$\log \left( {\widehat{Deaths}_{t} + 1} \right) = - 1.8986 + 0.8960* {\text{log}}\,(Cases_{t - 9} )$$
- City of New York:
  7 $$\log \left( {\widehat{Deaths}_{t} + 1} \right) = - 0.2840 + 0.7627* {\text{log}}\,(Cases_{t - 7} + 1)$$

For the city of New York, the DEM offers a 7-day window, while for the State of California the prediction window is nine days, the same as for the US as a whole. As regards the delayed elasticity parameters, we found a delayed elasticity of 0.896 for California, which is higher than for the US, which presents a value of 0.7839, while New York city shows a lower delayed elasticity, whose value is 0.7627. Finally, both the California and New York city models present an R-square of 0.98, similar to the 0.98 shown by the US model.

As already pointed out, the aim of the DEM is to fill the predictive gaps in the early stages of the pandemic. However, its predictive accuracy holds in the long-run. Indeed, the initial estimates of this work were performed on April 2. However, since the date of this study’s revision is early October, we already have enough observations available to evaluate its predictive revision accuracy in the long-run.

**Figure 2:**
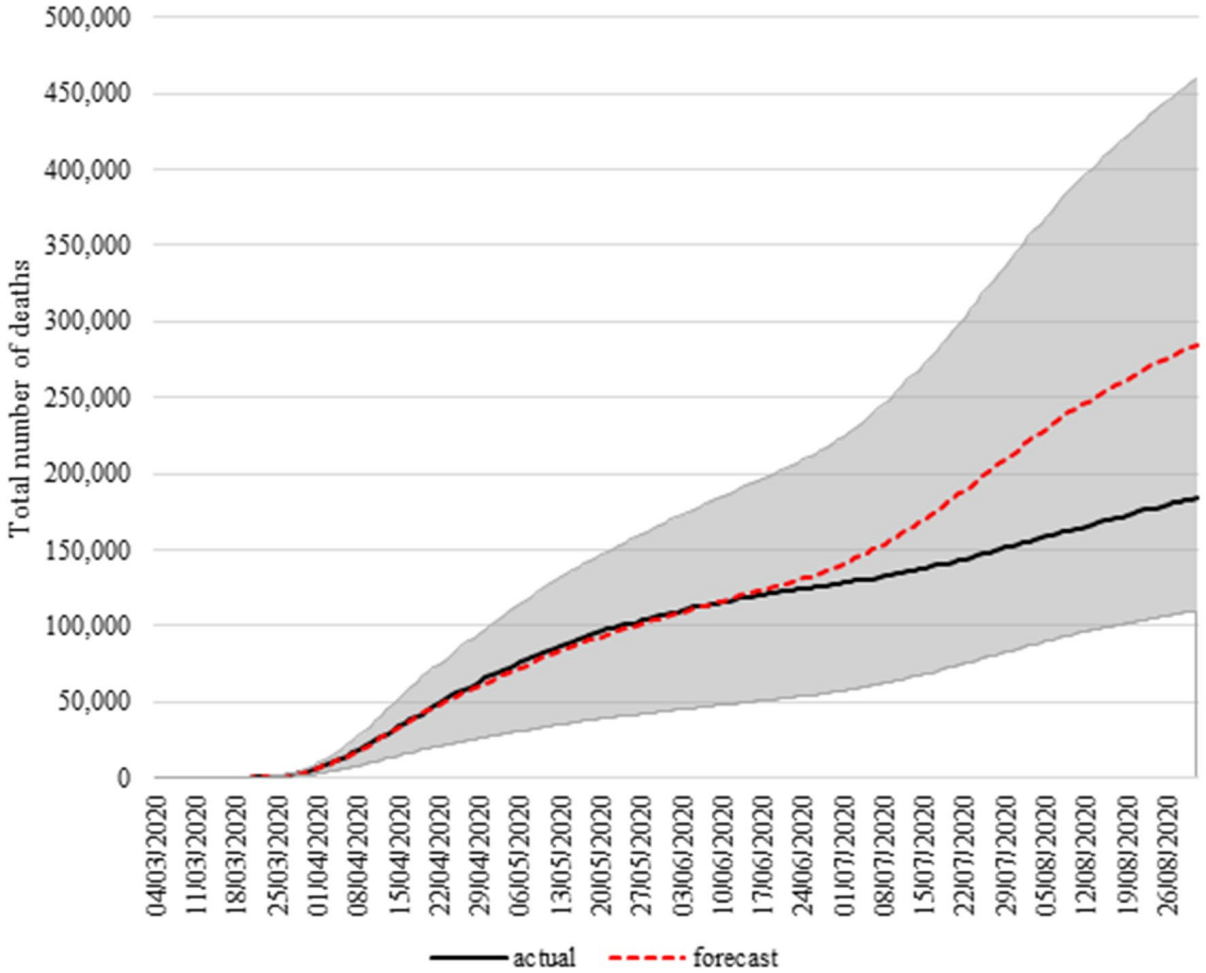
Actual vs estimated total COVID-19 deaths in the long-run for the US. *Source*: Authors’ own compilation and Johns Hopkins University CSSE (retrieved on 05/10/2020).

Figure 2 displays actual and estimated COVID-19 deaths extended up to August 31, 2020 (data extracted from Johns Hopkins University CSSE as of October 5, 2020 is included in Table C1 in the annex). As can be seen, the model estimates remain in an error range of below 10% until July 1: that is, we are able to estimate with a high degree of accuracy for just over three months, having used only 26 observations to obtain the representative equation for the prediction.

In sum, the results show that in the case of the US the deaths for the following nine days could have been predicted with a high level of accuracy during the expansive stage of the pandemic. The results also show that the DEM can be applied to different territorial levels, so that in the case of California, the predictions would also be nine days in advance, and seven days in New York city. We have also verified that the model remains stable over three months. In other words, without re-estimating the model, we could have maintained the same equation during the whole expansive stage of the pandemic to make the predictions.

## Discussion

The DEM is a model that does not require long time series and is therefore easily applicable in the early stages of the pandemic, when there is a lack of available data and when authorities need urgent and reliable predictions to fill the gap between the clinical needs caused by the pandemic and the available resources. In addition, it provides a prediction window, which in our case is nine days for deaths from Covid-19 in the US.

The DEM is applicable to other areas, as we have shown at the state and city level. It is also applicable by age groups if data for both variables are available. Obviously, disaggregation, as we have shown, must provide different delayed elasticities given that the pandemic does not evolve in the same way in different locations, and that the disease does not affect different age groups in the same way. Moreover, the DEM is versatile and can be applied to different types of clinical needs associated with the pandemic, such as hospitalizations, admissions to ICUs or ventilator needs.

Together with the possibilities indicated above, the DEM evidences certain difficulties that must be taken into account. First, the model is sensitive to any factor that affects the counts of both variables. For example, in the first stage of the Covid-19 pandemic, the health system did not have the means to perform all the tests needed, such that the model will clearly lose accuracy as testing capacity improves. There might also be differences and/or changes in the death count. States or territories may also define deaths differently, which will affect the comparability of the results, added to which the authorities might alter the criteria for counting deaths, which would imply greater model inaccuracy. Second, we must also take into account changes of all kinds that affect the relationship between infections and deaths during the evolution of the pandemic. This involves issues such as: the collapse of health systems, changes in treatments, or clinical innovations, changes in environmental factors or mutations of the virus that may alter its lethality, changes in the characteristics of the infected population, such as in the age pyramid of those infected… These factors make it advisable to use short time series and the closest observations in time, so that the above-mentioned changes are kept to a minimum.

Given that all the aforementioned factors may alter the accuracy of the model throughout the pandemic, it will need to be recalculated. To do this, in addition to estimating the model each time we become aware of any of the previously mentioned special circumstances, we can also establish systematic recalculation criteria such as: recalculating when the model presents a continuous prediction error above a certain percentage, for example 5% or 10%, or recalculating by setting a stable calendar, for example every week.

As stated, the DEM is linked to epidemiological models because it estimates the relationship between two epidemiological variables. For this reason, it opens up possibilities for further research on the relationship between delayed elasticity and the parameters of epidemiological models. Clarifying this possible relationship could allow the DEM to be integrated into epidemiological models in order to improve the latter’s predictive capacity. Due to its simplicity, versatility and predictive accuracy, the DEM can be applied to make predictions in other areas of clinical care where there are related cause-effect variables. This would substantially expand the research field of the methodology presented in this paper.

## Supplementary information

Supplementary Information.

## Data Availability

All data generated or analysed during this study are included in this published article (and its Supplementary Information files).
