# Supplementary material for: Predicting mortality for Covid-19 in the US using the delayed elasticity method
Source: Sci Rep. 2020 Nov 30;10:20811. doi: 10.1038/s41598-020-76490-8 (PMC7704650; doi:10.1038/s41598-020-76490-8)
Supplement: Supplementary file 1 — Supplementary Information. [file 41598_2020_76490_MOESM1_ESM.docx]

**Predicting mortality for Covid-19 in the US using the Delayed Elasticity Method**

**(Additional information)**

**Luis Ángel Hierro**

Senior Lecturer in Economics

Department of Economics and Economic History (Former Head)

Universidad de Sevilla

Avda. Ramón y Cajal, 1

41018 Sevilla (Spain)

Email: [lhierro@us.es](mailto:lhierro@us.es)

**Antonio J. Garzón**

Postgraduate Teaching Assistant in Economics

Department of Economics and Economic History

Universidad de Sevilla

Avda. Ramón y Cajal, 1

41018 Sevilla (Spain)

Email: [aggordon@us.es](mailto:aggordon@us.es)

**Pedro Atienza-Montero (Corresponding Author)**

Lecturer in Economics

Department of Economics and Economic History

Universidad de Sevilla

Avda. Ramón y Cajal, 1

41018 Sevilla (Spain)

Email: [atienza@us.es](mailto:atienza@us.es)

**José Luis Márquez**

Medical Doctor (Ph. Dr.)

Gastroenterology Unit (Former Head)

Hospital Universitario Virgen del Rocio

Avda. Manuel Siurot s/n

41013-Sevilla (Spain)

Email: [jlmarquezhuvr@gmail.com](mailto:jlmarquezhuvr@gmail.com)

1. **Other forecast performance indicators:**

Mean Absolute Error (MAE):

|  | $MAE= \frac{\sum_{t=T+1}^{T+N} \left\vert\hat{y_{t}}-y_{t} \right\vert}{N}$ | (2A) |
| --- | --- | --- |

Mean Absolute Percentage Error (MAPE):

|  | $MSE= \frac{{\sum_{t=T+1}^{T+N} (\hat{y_{t}}-y_{t})}^{2}}{N}$ | (3A) |
| --- | --- | --- |

Symmetric Mean Absolute Percentage Error (SMAPE):

|  | $SMAPE= \frac{\sum_{t=T+1}^{T+N} \left\vert{\hat{y_{t}}-y_{t}}/{y_{t}} \right\vert}{{(\left\vert y_{t} \right\vert+\left\vert\hat{y}_{t} \right\vert)}/2} x \frac{100}{N}$ | (4A) |
| --- | --- | --- |

1. **Out-of-sample forecast performance**

**Table A.1**. Out-of-sample forecast performance for COVID-19 deaths in the US

| Lags | RMSE | MAE | MAPE | SMAPE |
| --- | --- | --- | --- | --- |
| 1 | 2,453.27 | 2,367.49 | 44.06 | 56.74 |
| 2 | 2,262.8 | 2,171.45 | 40.26 | 50.66 |
| 3 | 2,084.3 | 1,996.14 | 36.96 | 45.57 |
| 4 | 1,864.43 | 1,788.49 | 33.15 | 39.89 |
| 5 | 1,693.9 | 1,633.17 | 30.38 | 35.91 |
| 6 | 1,423.19 | 1,361.7 | 25.18 | 28.91 |
| 7 | 1,049.44 | 972.46 | 17.65 | 19.49 |
| 8 | 515.9 | 472.85 | 8.56 | 8.98 |
| 9 | **74.66** | **53.52** | **0.91** | **0.9** |
| 10 | 685.53 | 662 | 12.42 | 11.69 |

Sources: authors’ own compilation. Selected lag in bold.

**Table A.2**. Out-of-sample forecast performance for COVID-19 deaths in the State of California

| Lags | RMSE | MAE | MAPE | SMAPE |
| --- | --- | --- | --- | --- |
| 1 | 27.56 | 25.04 | 15.67 | 17.3 |
| 2 | 24.98 | 22.8 | 13.9 | 15.17 |
| 3 | 19.76 | 19.15 | 11.61 | 12.37 |
| 4 | 17.01 | 14.54 | 9.09 | 9.72 |
| 5 | 21.42 | 20.04 | 12.41 | 13.29 |
| 6 | 19.77 | 18.76 | 11.52 | 12.21 |
| 7 | 18.7 | 17.25 | 10.75 | 11.42 |
| 8 | 17.99 | 17.64 | 10.61 | 10.69 |
| 9 | **14.38** | **11.45** | **6.87** | **6.53** |
| 10 | 41.03 | 39.06 | 23.42 | 20.81 |

Sources: authors’ own compilation. Selected lag in bold.

**Table A.3**. Out-of-sample forecast performance for COVID-19 deaths in the city of New York

| Lags | RMSE | MAE | MAPE | SMAPE |
| --- | --- | --- | --- | --- |
| 1 | 864.78 | 845.5 | 73.95 | 117.49 |
| 2 | 804.18 | 784.3 | 68.46 | 104.28 |
| 3 | 730.49 | 709.69 | 61.76 | 89.62 |
| 4 | 619.59 | 599.64 | 52.03 | 70.57 |
| 5 | 462.31 | 440.2 | 37.79 | 46.89 |
| 6 | 250.96 | 230.03 | 19.44 | 21.71 |
| 7 | **92.67** | **85.43** | **8.3** | **7.88** |
| 8 | 398.59 | 387.96 | 34.29 | 29.12 |
| 9 | 839.15 | 791.91 | 67.57 | 50.15 |
| 10 | 1,427.36 | 1,389.3 | 121.23 | 75.41 |

Sources: authors’ own compilation. Selected lag in bold.

1. **Estimation output for equation (1)**

**Table B1**. Estimation output for equation (1) in the US

| Variable | Coefficient | Std. Error | t-Statistic | Prob. |
| --- | --- | --- | --- | --- |
| LOG(CASES(-7)) | 0.783994 | 0.019852 | 39.489810 | 0.0000 |
| C | 0.369539 | 0.126050 | 2.931687 | 0.0073 |
| R-squared | 0.984843 | Mean dependent var | | 5.001085 |
| Adjusted R-squared | 0.984212 | S.D. dependent var | | 1.874170 |
| S.E. of regression | 0.235493 | Akaike info criterion | | 0.019530 |
| Sum squared resid | 1.330963 | Schwarz criterion | | 0.116306 |
| Log likelihood | 1.746113 | Hannan-Quinn criter. | | 0.047398 |
| F-statistic | 1559.445 | Durbin-Watson stat | | 0.381288 |
| Prob(F-statistic) | 0.000000 |  |  |  |

Source: authors’ own compilation

**Table B2**. Estimation output for equation (1) in the State of California

| Variable | Coefficient | Std. Error | t-Statistic | Prob. |
| --- | --- | --- | --- | --- |
| LOG(CASES(-9)) | 0.896003 | 0.027384 | 32.72039 | 0.0000 |
| C | -1.498607 | 0.127700 | -11.73539 | 0.0000 |
| R-squared | 0.978075 | Mean dependent var | | 2.429826 |
| Adjusted R-squared | 0.977161 | S.D. dependent var | | 1.467871 |
| S.E. of regression | 0.221833 | Akaike info criterion | | -0.099984 |
| Sum squared resid | 1.181033 | Schwarz criterion | | -0.003207 |
| Log likelihood | 3.299788 | Hannan-Quinn criter. | | -0.072115 |
| F-statistic | 1070.624 | Durbin-Watson stat | | 1.062092 |
| Prob(F-statistic) | 0.000000 |  |  |  |

Source: authors’ own compilation

**Table B3**. Estimation output for equation (1) in the city of New York

| Variable | Coefficient | Std. Error | t-Statistic | Prob. |
| --- | --- | --- | --- | --- |
| LOG(CASES(-7)+1) | 0.762732 | 0.019957 | 38.21928 | 0.0000 |
| C | -0.283951 | 0.096687 | -2.936801 | 0.0072 |
| R-squared | 0.983835 | Mean dependent var | | 2.493213 |
| Adjusted R-squared | 0.983162 | S.D. dependent var | | 2.506386 |
| S.E. of regression | 0.325235 | Akaike info criterion | | 0.665263 |
| Sum squared resid | 2.538660 | Schwarz criterion | | 0.762040 |
| Log likelihood | -6.648421 | Hannan-Quinn criter. | | 0.693131 |
| F-statistic | 1460.713 | Durbin-Watson stat | | 0.852521 |
| Prob(F-statistic) | 0.000000 |  |  |  |

Source: authors’ own compilation

1. **Model estimate values, actual values and residuals**

**Figure A1**. Model estimate values, actual values, and residuals for US (04/03/2020 – 29/03/2020)

Source: authors’ own compilation

**Figure A2.** Model estimate values, actual values, and residuals for the State of California (04/03/2020 – 29/03/2020)

Source: authors’ own compilation

**Figure A3.** Model estimate values, actual values, and residuals for the city of New York (04/03/2020 – 29/03/2020)

Source: authors’ own compilation

1. **Actual vs estimated total COVID-19 deaths**

**Figure B1**. Actual vs estimated total COVID-19 deaths in the US


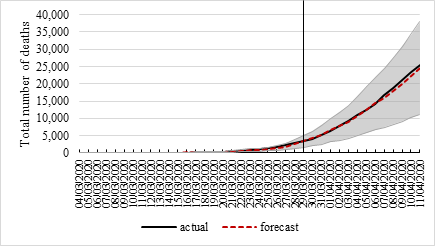


Source: authors’ own compilation and Johns Hopkins University CSSE (retrieved on 05/10/2020).

**Figure B2**. Actual vs estimated total COVID-19 deaths in the State of California


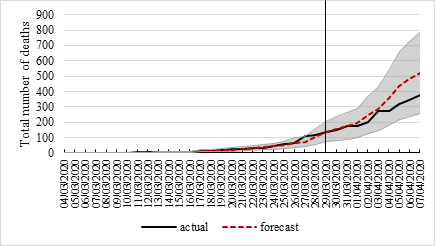


Source: authors’ own compilation and Johns Hopkins University CSSE (retrieved on 05/10/2020).

**Figure B3**. Actual vs estimated total COVID-19 deaths in the city of New York


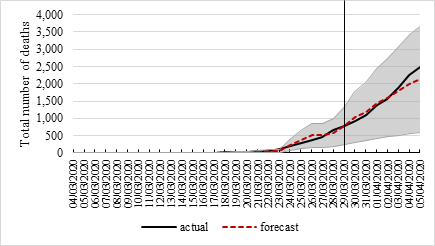


Source: authors’ own compilation and Johns Hopkins University CSSE (retrieved on 05/10/2020).

**Table C1.** Actual vs estimated COVID-19 deaths for the state of California

| Date | Cases | Deaths | Deaths (Est) | Error | Error rate |
| --- | --- | --- | --- | --- | --- |
| 04/03/2020 | 36 | 0 | 1 | 1 |  |
| 05/03/2020 | 45 | 1 | 1 | 0 | 24.14 |
| 06/03/2020 | 56 | 1 | 1 | 0 | 24.14 |
| 07/03/2020 | 56 | 1 | 1 | 0 | 8.46 |
| 08/03/2020 | 110 | 1 | 1 | 0 | 8.46 |
| 09/03/2020 | 135 | 2 | 1 | -1 | 46.47 |
| 10/03/2020 | 152 | 2 | 1 | -1 | 46.47 |
| 11/03/2020 | 175 | 3 | 2 | -1 | 19.37 |
| 12/03/2020 | 224 | 4 | 4 | 0 | 10.87 |
| 13/03/2020 | 264 | 4 | 5 | 1 | 13.53 |
| 14/03/2020 | 311 | 4 | 6 | 2 | 44.19 |
| 15/03/2020 | 369 | 5 | 7 | 2 | 44.66 |
| 16/03/2020 | 447 | 7 | 7 | 0 | 3.33 |
| 17/03/2020 | 596 | 10 | 14 | 4 | 40.75 |
| 18/03/2020 | 652 | 14 | 17 | 3 | 22.22 |
| 19/03/2020 | 982 | 18 | 19 | 1 | 6.34 |
| 20/03/2020 | 1,304 | 24 | 22 | -2 | 8.95 |
| 21/03/2020 | 1,534 | 27 | 28 | 1 | 1.89 |
| 22/03/2020 | 1,709 | 32 | 32 | 0 | 0.10 |
| 23/03/2020 | 1,931 | 35 | 37 | 2 | 6.44 |
| 24/03/2020 | 2,511 | 44 | 44 | 0 | 0.93 |
| 25/03/2020 | 2,982 | 57 | 52 | -5 | 8.86 |
| 26/03/2020 | 3,777 | 64 | 68 | 4 | 5.49 |
| 27/03/2020 | 4,730 | 107 | 73 | -34 | 31.54 |
| 28/03/2020 | 5,259 | 119 | 106 | -13 | 10.77 |
| 29/03/2020 | 5,739 | 137 | 137 | 0 | 0.14 |
| 30/03/2020 | **6,909** | **151** | **159** | **8** | **5.19** |
| 31/03/2020 | **8,131** | **172** | **175** | **3** | **1.79** |
| 01/04/2020 | **8,155** | **172** | **195** | **23** | **13.63** |
| 02/04/2020 | **9,191** | **203** | **248** | **45** | **21.95** |
| 03/04/2020 | **12,024** | **270** | **289** | **19** | **7.02** |
| 04/04/2020 | **12,026** | **276** | **357** | **81** | **29.47** |
| 05/04/2020 | **13,438** | **319** | **437** | **118** | **37.11** |
| 06/04/2020 | **14,336** | **343** | **481** | **138** | **40.25** |
| 07/04/2020 | **15,865** | **374** | **520** | **146** | **39.12** |

Source: authors’ own compilation and Johns Hopkins University CSSE (retrieved on 05/10/2020). Deaths(est) values are rounded to integer values. Out-of-sample dates in bold.

**Table C2.** Actual vs estimated COVID-19 deaths for the city of New York

| Date | Cases | Deaths | Deaths (Est) | Error | Error rate |
| --- | --- | --- | --- | --- | --- |
| 04/03/2020 | 1 | 0 | 0 | 0 |  |
| 05/03/2020 | 2 | 0 | 0 | 0 |  |
| 06/03/2020 | 2 | 0 | 0 | 0 |  |
| 07/03/2020 | 2 | 0 | 0 | 0 |  |
| 08/03/2020 | 12 | 0 | 0 | 0 |  |
| 09/03/2020 | 17 | 0 | 0 | 0 |  |
| 10/03/2020 | 36 | 0 | 0 | 0 |  |
| 11/03/2020 | 53 | 0 | 0 | 0 |  |
| 12/03/2020 | 95 | 0 | 1 | 1 |  |
| 13/03/2020 | 95 | 0 | 1 | 1 |  |
| 14/03/2020 | 150 | 0 | 1 | 1 |  |
| 15/03/2020 | 269 | 5 | 4 | -1 | 13.50 |
| 16/03/2020 | 344 | 5 | 6 | 1 | 16.50 |
| 17/03/2020 | 1,871 | 11 | 11 | 0 | 1.59 |
| 18/03/2020 | 3,554 | 21 | 15 | -6 | 29.63 |
| 19/03/2020 | 5,151 | 29 | 23 | -6 | 19.07 |
| 20/03/2020 | 5,151 | 29 | 23 | -6 | 19.07 |
| 21/03/2020 | 6,211 | 43 | 34 | -9 | 21.94 |
| 22/03/2020 | 9,045 | 63 | 53 | -10 | 16.12 |
| 23/03/2020 | 13,119 | 125 | 64 | -61 | 48.87 |
| 24/03/2020 | 15,597 | 192 | 235 | 43 | 22.30 |
| 25/03/2020 | 20,011 | 280 | 384 | 104 | 37.00 |
| 26/03/2020 | 23,112 | 365 | 509 | 144 | 39.57 |
| 27/03/2020 | 26,697 | 450 | 509 | 59 | 13.20 |
| 28/03/2020 | 30,756 | 672 | 588 | -84 | 12.54 |
| 29/03/2020 | 33,474 | 776 | 783 | 7 | 0.92 |
| 30/03/2020 | **38,087** | **914** | **1,040** | **126** | **13.81** |
| 31/03/2020 | **41,771** | **1,096** | **1,187** | **91** | **8.32** |
| 01/04/2020 | **48,462** | **1,397** | **1,436** | **39** | **2.78** |
| 02/04/2020 | **49,707** | **1,562** | **1,603** | **41** | **2.61** |
| 03/04/2020 | **56,289** | **1,867** | **1,789** | **-78** | **4.17** |
| 04/04/2020 | **60,850** | **2,254** | **1,993** | **-261** | **11.57** |
| 05/04/2020 | **64,955** | **2,472** | **2,126** | **-346** | **13.98** |

Source: authors’ own compilation and Johns Hopkins University CSSE (retrieved on 05/10/2020). Deaths(est) values are rounded to integer values. Out-of-sample dates in bold.

**Table C3.** Actual vs estimated COVID-19 deaths for the US in the long-run (04/03/2020 – 28/09/2020)

| Date | Cases | Deaths | Deaths (Est) | Error | Error rate |
| --- | --- | --- | --- | --- | --- |
| 04/03/2020 | 107 | 11 | 13 | 2 | 0.16 |
| 05/03/2020 | 184 | 12 | 13 | 1 | 0.06 |
| 06/03/2020 | 237 | 14 | 13 | -1 | 0.09 |
| 07/03/2020 | 403 | 17 | 13 | -4 | 0.22 |
| 08/03/2020 | 519 | 21 | 13 | -8 | 0.36 |
| 09/03/2020 | 594 | 22 | 18 | -4 | 0.18 |
| 10/03/2020 | 782 | 28 | 22 | -6 | 0.22 |
| 11/03/2020 | 1,147 | 33 | 33 | 0 | 0.01 |
| 12/03/2020 | 1,586 | 43 | 42 | -1 | 0.02 |
| 13/03/2020 | 2,219 | 51 | 56 | 5 | 0.11 |
| 14/03/2020 | 2,978 | 58 | 86 | 28 | 0.49 |
| 15/03/2020 | 3,212 | 70 | 105 | 35 | 0.50 |
| 16/03/2020 | 4,679 | 97 | 160 | 63 | 0.64 |
| 17/03/2020 | 6,511 | 132 | 195 | 63 | 0.47 |
| 18/03/2020 | 9,165 | 191 | 216 | 25 | 0.13 |
| 19/03/2020 | 13,659 | 265 | 268 | 3 | 0.01 |
| 20/03/2020 | 20,026 | 364 | 362 | -2 | 0.00 |
| 21/03/2020 | 26,022 | 463 | 467 | 4 | 0.01 |
| 22/03/2020 | 34,824 | 573 | 608 | 35 | 0.06 |
| 23/03/2020 | 46,043 | 762 | 765 | 3 | 0.00 |
| 24/03/2020 | 56,620 | 1,001 | 812 | -189 | 0.19 |
| 25/03/2020 | 68,654 | 1,325 | 1,091 | -234 | 0.18 |
| 26/03/2020 | 86,548 | 1,733 | 1,413 | -320 | 0.18 |
| 27/03/2020 | 105,179 | 2,253 | 1,847 | -406 | 0.18 |
| 28/03/2020 | 124,786 | 2,886 | 2,526 | -360 | 0.12 |
| 29/03/2020 | 143,715 | 3,472 | 3,409 | -63 | 0.02 |
| 30/03/2020 | **165,728** | **4,164** | **4,186** | **22** | **0.01** |
| 31/03/2020 | **192,091** | **5,249** | **5,260** | **11** | **0.00** |
| 01/04/2020 | **217,910** | **6,421** | **6,548** | **127** | **0.02** |
| 02/04/2020 | **248,302** | **7,924** | **7,700** | **-224** | **0.03** |
| 03/04/2020 | **280,302** | **9,316** | **8,956** | **-360** | **0.04** |
| 04/04/2020 | **313,303** | **10,839** | **10,739** | **-100** | **0.01** |
| 05/04/2020 | **341,487** | **12,429** | **12,513** | **84** | **0.01** |
| 06/04/2020 | **371,672** | **14,199** | **14,307** | **108** | **0.01** |
| 07/04/2020 | **403,071** | **16,770** | **15,982** | **-788** | **0.05** |
| 08/04/2020 | **435,087** | **18,916** | **17,871** | **-1,045** | **0.06** |
| 09/04/2020 | **469,735** | **21,144** | **20,064** | **-1,080** | **0.05** |
| 10/04/2020 | **503,271** | **23,362** | **22,149** | **-1,213** | **0.05** |
| 11/04/2020 | **532,628** | **25,481** | **24,536** | **-945** | **0.04** |
| 12/04/2020 | **559,587** | **27,294** | **26,982** | **-312** | **0.01** |
| 13/04/2020 | **585,378** | **29,240** | **29,442** | **202** | **0.01** |
| 14/04/2020 | **613,969** | **31,695** | **31,498** | **-197** | **0.01** |
| 15/04/2020 | **643,977** | **34,304** | **33,661** | **-643** | **0.02** |
| 16/04/2020 | **675,499** | **36,480** | **35,871** | **-609** | **0.02** |
| 17/04/2020 | **708,305** | **38,573** | **38,086** | **-487** | **0.01** |
| 18/04/2020 | **736,156** | **40,533** | **40,443** | **-90** | **0.00** |
| 19/04/2020 | **761,837** | **42,464** | **42,690** | **226** | **0.01** |
| 20/04/2020 | **790,259** | **44,650** | **44,630** | **-20** | **0.00** |
| 21/04/2020 | **816,297** | **47,198** | **46,391** | **-807** | **0.02** |
| 22/04/2020 | **845,594** | **49,632** | **48,059** | **-1,573** | **0.03** |
| 23/04/2020 | **879,025** | **52,101** | **49,890** | **-2,211** | **0.04** |
| 24/04/2020 | **912,591** | **54,255** | **51,792** | **-2,463** | **0.05** |
| 25/04/2020 | **944,392** | **55,953** | **53,769** | **-2,184** | **0.04** |
| 26/04/2020 | **971,491** | **57,282** | **55,805** | **-1,477** | **0.03** |
| 27/04/2020 | **994,665** | **58,748** | **57,518** | **-1,230** | **0.02** |
| 28/04/2020 | **1,019,339** | **60,989** | **59,085** | **-1,904** | **0.03** |
| 29/04/2020 | **1,047,184** | **63,510** | **60,807** | **-2,703** | **0.04** |
| 30/04/2020 | **1,076,689** | **65,832** | **62,372** | **-3,460** | **0.05** |
| 01/05/2020 | **1,110,909** | **67,723** | **64,120** | **-3,603** | **0.05** |
| 02/05/2020 | **1,138,911** | **69,415** | **66,099** | **-3,316** | **0.05** |
| 03/05/2020 | **1,163,413** | **70,535** | **68,069** | **-2,466** | **0.03** |
| 04/05/2020 | **1,186,815** | **71,868** | **69,922** | **-1,946** | **0.03** |
| 05/05/2020 | **1,211,378** | **74,186** | **71,490** | **-2,696** | **0.04** |
| 06/05/2020 | **1,236,525** | **76,550** | **72,823** | **-3,727** | **0.05** |
| 07/05/2020 | **1,264,315** | **78,484** | **74,236** | **-4,248** | **0.05** |
| 08/05/2020 | **1,291,197** | **80,231** | **75,821** | **-4,410** | **0.05** |
| 09/05/2020 | **1,316,165** | **81,719** | **77,491** | **-4,228** | **0.05** |
| 10/05/2020 | **1,335,021** | **82,612** | **79,415** | **-3,197** | **0.04** |
| 11/05/2020 | **1,354,468** | **83,626** | **80,980** | **-2,646** | **0.03** |
| 12/05/2020 | **1,377,226** | **85,253** | **82,342** | **-2,911** | **0.03** |
| 13/05/2020 | **1,398,192** | **87,017** | **83,638** | **-3,379** | **0.04** |
| 14/05/2020 | **1,425,500** | **88,800** | **84,992** | **-3,808** | **0.04** |
| 15/05/2020 | **1,450,798** | **90,481** | **86,372** | **-4,109** | **0.05** |
| 16/05/2020 | **1,474,811** | **91,697** | **87,890** | **-3,807** | **0.04** |
| 17/05/2020 | **1,492,925** | **92,455** | **89,352** | **-3,103** | **0.03** |
| 18/05/2020 | **1,514,937** | **93,627** | **90,703** | **-2,924** | **0.03** |
| 19/05/2020 | **1,535,981** | **95,179** | **91,721** | **-3,458** | **0.04** |
| 20/05/2020 | **1,559,486** | **96,705** | **92,766** | **-3,939** | **0.04** |
| 21/05/2020 | **1,585,151** | **97,922** | **93,986** | **-3,936** | **0.04** |
| 22/05/2020 | **1,608,589** | **99,166** | **95,106** | **-4,060** | **0.04** |
| 23/05/2020 | **1,629,786** | **100,275** | **96,559** | **-3,716** | **0.04** |
| 24/05/2020 | **1,649,900** | **100,897** | **97,900** | **-2,997** | **0.03** |
| 25/05/2020 | **1,668,217** | **101,445** | **99,168** | **-2,277** | **0.02** |
| 26/05/2020 | **1,687,741** | **102,107** | **100,121** | **-1,986** | **0.02** |
| 27/05/2020 | **1,706,331** | **103,625** | **101,277** | **-2,348** | **0.02** |
| 28/05/2020 | **1,729,279** | **104,803** | **102,378** | **-2,425** | **0.02** |
| 29/05/2020 | **1,753,630** | **105,963** | **103,604** | **-2,359** | **0.02** |
| 30/05/2020 | **1,777,473** | **106,926** | **104,938** | **-1,988** | **0.02** |
| 31/05/2020 | **1,796,645** | **107,507** | **106,153** | **-1,354** | **0.01** |
| 01/06/2020 | **1,814,009** | **108,283** | **107,248** | **-1,035** | **0.01** |
| 02/06/2020 | **1,835,382** | **109,351** | **108,284** | **-1,067** | **0.01** |
| 03/06/2020 | **1,855,359** | **110,358** | **109,225** | **-1,133** | **0.01** |
| 04/06/2020 | **1,877,078** | **111,381** | **110,226** | **-1,155** | **0.01** |
| 05/06/2020 | **1,902,245** | **112,295** | **111,177** | **-1,118** | **0.01** |
| 06/06/2020 | **1,924,081** | **112,954** | **112,347** | **-607** | **0.01** |
| 07/06/2020 | **1,941,868** | **113,392** | **113,586** | **194** | **0.00** |
| 08/06/2020 | **1,959,396** | **113,911** | **114,795** | **884** | **0.01** |
| 09/06/2020 | **1,977,765** | **114,861** | **115,764** | **903** | **0.01** |
| 10/06/2020 | **1,998,589** | **115,762** | **116,640** | **878** | **0.01** |
| 11/06/2020 | **2,021,786** | **116,633** | **117,716** | **1,083** | **0.01** |
| 12/06/2020 | **2,046,651** | **117,461** | **118,720** | **1,259** | **0.01** |
| 13/06/2020 | **2,071,911** | **118,220** | **119,808** | **1,588** | **0.01** |
| 14/06/2020 | **2,091,283** | **118,528** | **121,065** | **2,537** | **0.02** |
| 15/06/2020 | **2,111,207** | **118,922** | **122,153** | **3,231** | **0.03** |
| 16/06/2020 | **2,135,002** | **119,760** | **123,038** | **3,278** | **0.03** |
| 17/06/2020 | **2,160,906** | **120,504** | **123,907** | **3,403** | **0.03** |
| 18/06/2020 | **2,189,091** | **121,222** | **124,817** | **3,595** | **0.03** |
| 19/06/2020 | **2,220,401** | **121,892** | **125,846** | **3,954** | **0.03** |
| 20/06/2020 | **2,252,655** | **122,496** | **126,990** | **4,494** | **0.04** |
| 21/06/2020 | **2,278,761** | **122,781** | **128,212** | **5,431** | **0.04** |
| 22/06/2020 | **2,309,243** | **123,170** | **129,451** | **6,281** | **0.05** |
| 23/06/2020 | **2,345,008** | **124,017** | **130,399** | **6,382** | **0.05** |
| 24/06/2020 | **2,379,473** | **124,776** | **131,372** | **6,596** | **0.05** |
| 25/06/2020 | **2,420,009** | **125,327** | **132,531** | **7,204** | **0.06** |
| 26/06/2020 | **2,465,323** | **125,944** | **133,790** | **7,846** | **0.06** |
| 27/06/2020 | **2,507,672** | **126,448** | **135,156** | **8,708** | **0.07** |
| 28/06/2020 | **2,547,023** | **126,721** | **136,670** | **9,949** | **0.08** |
| 29/06/2020 | **2,587,708** | **127,098** | **138,223** | **11,125** | **0.09** |
| 30/06/2020 | **2,633,871** | **127,723** | **139,478** | **11,755** | **0.09** |
| 01/07/2020 | **2,685,191** | **128,417** | **140,938** | **12,521** | **0.10** |
| 02/07/2020 | **2,740,301** | **129,144** | **142,646** | **13,502** | **0.10** |
| 03/07/2020 | **2,792,849** | **129,804** | **144,287** | **14,483** | **0.11** |
| 04/07/2020 | **2,838,589** | **130,069** | **146,211** | **16,142** | **0.12** |
| 05/07/2020 | **2,888,566** | **130,357** | **148,353** | **17,996** | **0.14** |
| 06/07/2020 | **2,933,216** | **130,712** | **150,347** | **19,635** | **0.15** |
| 07/07/2020 | **2,993,372** | **131,934** | **152,193** | **20,259** | **0.15** |
| 08/07/2020 | **3,052,545** | **132,797** | **154,096** | **21,299** | **0.16** |
| 09/07/2020 | **3,115,816** | **133,800** | **156,247** | **22,447** | **0.17** |
| 10/07/2020 | **3,183,535** | **134,624** | **158,628** | **24,004** | **0.18** |
| 11/07/2020 | **3,243,686** | **135,314** | **161,175** | **25,861** | **0.19** |
| 12/07/2020 | **3,302,565** | **135,773** | **163,593** | **27,820** | **0.20** |
| 13/07/2020 | **3,362,035** | **136,140** | **165,689** | **29,549** | **0.22** |
| 14/07/2020 | **3,428,992** | **137,066** | **167,972** | **30,906** | **0.23** |
| 15/07/2020 | **3,496,615** | **138,034** | **170,004** | **31,970** | **0.23** |
| 16/07/2020 | **3,573,977** | **138,977** | **172,731** | **33,754** | **0.24** |
| 17/07/2020 | **3,645,987** | **139,907** | **175,402** | **35,495** | **0.25** |
| 18/07/2020 | **3,708,735** | **140,775** | **178,246** | **37,471** | **0.27** |
| 19/07/2020 | **3,769,990** | **141,222** | **181,276** | **40,054** | **0.28** |
| 20/07/2020 | **3,831,932** | **141,736** | **183,956** | **42,220** | **0.30** |
| 21/07/2020 | **3,896,363** | **142,843** | **186,568** | **43,725** | **0.31** |
| 22/07/2020 | **3,967,668** | **144,059** | **189,197** | **45,138** | **0.31** |
| 23/07/2020 | **4,036,741** | **145,153** | **192,144** | **46,991** | **0.32** |
| 24/07/2020 | **4,109,848** | **146,285** | **195,109** | **48,824** | **0.33** |
| 25/07/2020 | **4,176,302** | **147,184** | **198,485** | **51,301** | **0.35** |
| 26/07/2020 | **4,231,104** | **147,656** | **201,613** | **53,957** | **0.37** |
| 27/07/2020 | **4,287,684** | **148,776** | **204,328** | **55,552** | **0.37** |
| 28/07/2020 | **4,353,477** | **150,150** | **206,969** | **56,819** | **0.38** |
| 29/07/2020 | **4,424,779** | **151,582** | **209,630** | **58,048** | **0.38** |
| 30/07/2020 | **4,492,558** | **152,791** | **212,388** | **59,597** | **0.39** |
| 31/07/2020 | **4,559,711** | **154,034** | **215,429** | **61,395** | **0.40** |
| 01/08/2020 | **4,617,614** | **155,149** | **218,364** | **63,215** | **0.41** |
| 02/08/2020 | **4,664,943** | **155,552** | **221,458** | **65,906** | **0.42** |
| 03/08/2020 | **4,709,773** | **156,082** | **224,260** | **68,178** | **0.44** |
| 04/08/2020 | **4,767,233** | **157,460** | **226,564** | **69,104** | **0.44** |
| 05/08/2020 | **4,820,885** | **158,836** | **228,936** | **70,100** | **0.44** |
| 06/08/2020 | **4,880,525** | **160,089** | **231,685** | **71,596** | **0.45** |
| 07/08/2020 | **4,938,857** | **161,331** | **234,654** | **73,323** | **0.45** |
| 08/08/2020 | **4,994,547** | **162,408** | **237,468** | **75,060** | **0.46** |
| 09/08/2020 | **5,040,670** | **162,919** | **240,246** | **77,327** | **0.47** |
| 10/08/2020 | **5,090,430** | **163,446** | **242,634** | **79,188** | **0.48** |
| 11/08/2020 | **5,137,333** | **164,510** | **244,582** | **80,072** | **0.49** |
| 12/08/2020 | **5,194,337** | **166,015** | **246,422** | **80,407** | **0.48** |
| 13/08/2020 | **5,246,314** | **167,085** | **248,776** | **81,691** | **0.49** |
| 14/08/2020 | **5,310,915** | **168,423** | **250,968** | **82,545** | **0.49** |
| 15/08/2020 | **5,358,340** | **169,457** | **253,399** | **83,942** | **0.50** |
| 16/08/2020 | **5,399,753** | **170,028** | **255,770** | **85,742** | **0.50** |
| 17/08/2020 | **5,435,692** | **170,474** | **258,028** | **87,554** | **0.51** |
| 18/08/2020 | **5,481,046** | **171,799** | **259,894** | **88,095** | **0.51** |
| 19/08/2020 | **5,528,376** | **173,152** | **261,903** | **88,751** | **0.51** |
| 20/08/2020 | **5,572,457** | **174,231** | **263,793** | **89,562** | **0.51** |
| 21/08/2020 | **5,620,676** | **175,336** | **266,085** | **90,749** | **0.52** |
| 22/08/2020 | **5,665,086** | **176,318** | **268,170** | **91,852** | **0.52** |
| 23/08/2020 | **5,699,515** | **176,768** | **270,755** | **93,987** | **0.53** |
| 24/08/2020 | **5,737,369** | **177,210** | **272,649** | **95,439** | **0.54** |
| 25/08/2020 | **5,775,598** | **178,449** | **274,300** | **95,851** | **0.54** |
| 26/08/2020 | **5,820,689** | **179,674** | **275,730** | **96,056** | **0.53** |
| 27/08/2020 | **5,866,560** | **180,785** | **277,532** | **96,747** | **0.54** |
| 28/08/2020 | **5,912,756** | **181,756** | **279,409** | **97,653** | **0.54** |
| 29/08/2020 | **5,958,776** | **182,714** | **281,154** | **98,440** | **0.54** |
| 30/08/2020 | **5,994,130** | **183,024** | **283,059** | **100,035** | **0.55** |
| 31/08/2020 | **6,028,642** | **183,597** | **284,811** | **101,214** | **0.55** |
| 01/09/2020 | **6,071,171** | **184,664** | **286,167** | **101,503** | **0.55** |
| 02/09/2020 | **6,111,774** | **185,720** | **287,656** | **101,936** | **0.55** |
| 03/09/2020 | **6,155,692** | **186,790** | **289,157** | **102,367** | **0.55** |
| 04/09/2020 | **6,205,799** | **187,755** | **290,925** | **103,170** | **0.55** |
| 05/09/2020 | **6,249,850** | **188,538** | **292,721** | **104,183** | **0.55** |
| 06/09/2020 | **6,280,981** | **188,941** | **294,527** | **105,586** | **0.56** |
| 07/09/2020 | **6,305,037** | **189,208** | **296,322** | **107,114** | **0.57** |
| 08/09/2020 | **6,331,773** | **189,653** | **297,700** | **108,047** | **0.57** |
| 09/09/2020 | **6,365,325** | **190,846** | **299,042** | **108,196** | **0.57** |
| 10/09/2020 | **6,401,685** | **191,766** | **300,695** | **108,929** | **0.57** |
| 11/09/2020 | **6,449,207** | **192,979** | **302,270** | **109,291** | **0.57** |
| 12/09/2020 | **6,490,330** | **193,693** | **303,972** | **110,279** | **0.57** |
| 13/09/2020 | **6,524,954** | **194,071** | **305,910** | **111,839** | **0.58** |
| 14/09/2020 | **6,558,900** | **194,493** | **307,611** | **113,118** | **0.58** |
| 15/09/2020 | **6,598,091** | **195,781** | **308,811** | **113,030** | **0.58** |
| 16/09/2020 | **6,636,642** | **196,764** | **309,738** | **112,974** | **0.57** |
| 17/09/2020 | **6,681,372** | **197,634** | **310,767** | **113,133** | **0.57** |
| 18/09/2020 | **6,730,088** | **198,570** | **312,057** | **113,487** | **0.57** |
| 19/09/2020 | **6,774,138** | **199,282** | **313,454** | **114,172** | **0.57** |
| 20/09/2020 | **6,810,352** | **199,509** | **315,277** | **115,768** | **0.58** |
| 21/09/2020 | **6,862,816** | **199,865** | **316,851** | **116,986** | **0.59** |
| 22/09/2020 | **6,901,781** | **200,786** | **318,176** | **117,390** | **0.58** |
| 23/09/2020 | **6,940,214** | **201,884** | **319,473** | **117,589** | **0.58** |
| 24/09/2020 | **6,984,346** | **202,798** | **320,968** | **118,170** | **0.58** |
| 25/09/2020 | **7,034,931** | **203,750** | **322,437** | **118,687** | **0.58** |
| 26/09/2020 | **7,079,803** | **204,490** | **324,140** | **119,650** | **0.59** |
| 27/09/2020 | **7,116,225** | **204,756** | **325,991** | **121,235** | **0.59** |
| 28/09/2020 | **7,149,537** | **205,072** | **327,663** | **122,591** | **0.60** |

Source: authors’ own compilation and Johns Hopkins University CSSE (retrieved on 05/10/2020). Deaths(est) values are rounded to integer values. Out-of-sample dates in bold.
